# Niemann-Pick disease type C

**DOI:** 10.1186/1750-1172-5-16

**Published:** 2010-06-03

**Authors:** Marie T Vanier

**Affiliations:** 1 Institut National de la Santé et de la Recherche Médicale, Unité 820, Faculté de Médecine Lyon-Est Claude Bernard, 7 Rue G. Paradin, F-69008, Lyon, France; 2 Hospices Civils de Lyon, Laboratoire de Neurobiologie Gillet-Mérieux, Centre de Biologie Est, 59 Bd Pinel, F-69500, Bron, France

## Abstract

Niemann-Pick C disease (NP-C) is a neurovisceral atypical lysosomal lipid storage disorder with an estimated minimal incidence of 1/120 000 live births. The broad clinical spectrum ranges from a neonatal rapidly fatal disorder to an adult-onset chronic neurodegenerative disease. The neurological involvement defines the disease severity in most patients but is typically preceded by systemic signs (cholestatic jaundice in the neonatal period or isolated spleno- or hepatosplenomegaly in infancy or childhood). The first neurological symptoms vary with age of onset: delay in developmental motor milestones (early infantile period), gait problems, falls, clumsiness, cataplexy, school problems (late infantile and juvenile period), and ataxia not unfrequently following initial psychiatric disturbances (adult form). The most characteristic sign is vertical supranuclear gaze palsy. The neurological disorder consists mainly of cerebellar ataxia, dysarthria, dysphagia, and progressive dementia. Cataplexy, seizures and dystonia are other common features. NP-C is transmitted in an autosomal recessive manner and is caused by mutations of either the *NPC1 *(95% of families) or the *NPC2 *genes. The exact functions of the NPC1 and NPC2 proteins are still unclear. NP-C is currently described as a cellular cholesterol trafficking defect but in the brain, the prominently stored lipids are gangliosides. Clinical examination should include comprehensive neurological and ophthalmological evaluations. The primary laboratory diagnosis requires living skin fibroblasts to demonstrate accumulation of unesterified cholesterol in perinuclear vesicles (lysosomes) after staining with filipin. Pronounced abnormalities are observed in about 80% of the cases, mild to moderate alterations in the remainder ("variant" biochemical phenotype). Genotyping of patients is useful to confirm the diagnosis in the latter patients and essential for future prenatal diagnosis. The differential diagnosis may include other lipidoses; idiopathic neonatal hepatitis and other causes of cholestatic icterus should be considered in neonates, and conditions with cerebellar ataxia, dystonia, cataplexy and supranuclear gaze palsy in older children and adults. Symptomatic management of patients is crucial. A first product, miglustat, has been granted marketing authorization in Europe and several other countries for specific treatment of the neurological manifestations. The prognosis largely correlates with the age at onset of the neurological manifestations.

## Disease definition

### Historical delineation

Coined in the late 1920's from the pioneering work of Albert Niemann and Ludwig Pick, the eponym "Niemann-Pick disease" has since been used to designate a heterogeneous group of autosomal recessive lysosomal lipid storage disorders, with common features of hepatosplenomegaly and sphingomyelin storage in reticuloendothelial and parenchymal tissues, with or without neurological involvement. In 1958, Crocker and Farber showed that there was a wide variability in age of onset and clinical expression, as well as in the level of sphingomyelin storage in tissues [1]. This led Crocker to propose a classification into four subgroups, A to D [2]. Type A was characterized by severe, early CNS deterioration and massive visceral and cerebral sphingomyelin storage. Type B showed a chronic course with marked visceral involvement but a sparing of the nervous system. Types C and D were characterized by a sub acute nervous system involvement with a moderate and slower course and a milder visceral storage. Type D patients were individualized essentially on their homogenous Nova Scotia Acadian origin. In 1966, Brady and associates [3] demonstrated a severe deficiency in sphingomyelinase activity in tissues from patients with type A, a finding soon extended to type B, but not to types C and D, indicating that the two latter types constituted separate entities. From that time on, with a turn following seminal observations in a Balb/c murine model of the disorder [4], the concept of Niemann-Pick type C disease evolved from that of a sphingomyelin storage disorder to that of a cholesterol storage disorder [5]. This and later work led to the reclassification of type C as a cellular lipid trafficking disorder, involving more specially, but not only, endocytosed cholesterol.

### Definition of Niemann-Pick disease type C

Today, by definition, "Niemann-Pick C disease" encompasses disorders characterized by unique abnormalities of intracellular transport of endocytosed cholesterol with sequestration of unesterified cholesterol in lysosomes and late endosomes [5-12]. Major advances have been the description of two genetic complementation groups [13,14] and the subsequent isolation of the two underlying genes [15,16]. *NPC1 *is involved in 95% of the families [14], including those with type D [17]. *NPC2 *is involved in rare families (about 30 are known to date). Although the precise functions of the NPC1 and NPC2 proteins are still elusive, current knowledge supports the idea that these proteins function in a coordinate fashion and that they are involved in the cellular postlysosomal/late endosomal transport of cholesterol and other molecules [10-12,18,19].

Niemann-Pick diseases thus oppose two clearly distinct groups: acid sphingomyelinase deficiencies (due to *SMPD1 *mutations, including types A, B and intermediate forms,) and Niemann-Pick type C, with alterations in trafficking of endocytosed cholesterol (due to *NPC1 *or *NPC2 *mutations). Type D as a distinct entity is no longer justified. From a practical standpoint, no patient should today be longer qualified of suffering from "Niemann-Pick disease" without specification of the subgroup, either acid sphingomyelinase deficiency or type C.

## Disease name and synonyms

"Niemann-Pick disease type C" (or "Niemann-Pick C disease"), often abbreviated as NP-C (or NPC), is currently the generic name widely used to designate the condition, irrespective of which gene, *NPC1 *or *NPC2*, is mutated. This term now encompasses the historical Niemann-Pick disease type D referring to the "Nova Scotia" isolate, later shown to be a genetic *NPC1 *variant [17]. Instead, a subdivision is sometimes made between Niemann-Pick C1 (NP-C1) or C2 (NP-C2), according to the gene involved. Patients with a retrospective diagnosis of Niemann-Pick C disease have also been described in the 1960s and 1970s as juvenile Niemann-Pick disease, juvenile dystonic lipidosis, atypical cerebral lipidosis, neurovisceral storage disease with vertical supranuclear ophthalmoplegia, maladie de Neville, DAF (down-gaze paresis, ataxia, foam cell) syndrome, adult dystonic lipidosis, adult neurovisceral lipidosis, giant cell hepatitis, and lactosylceramidosis [10,20].

## Epidemiology

NP-C (either NP-C1 or NP-C2) shows autosomal recessive inheritance and is panethnic. The true prevalence of NP-C is difficult to assess, because of insufficient clinical awareness combined with the relative difficulty of biochemical testing. Estimates of birth prevalence ranging between 0.66 and 0.83 per 100,000 were proposed for France, the UK and Germany based on diagnoses made in the laboratory of the author over the period 1988-2002 [10,11]. However, very different figures of 0.47, 0.35 and 2.20 per 100,000, respectively, were reported in studies from Australia (20 cases between 1980-1996), The Netherlands (25 cases between 1970-1996) and Northern Portugal (9 cases 1985-2003) [21-23]. The low incidence found for Australia and the Netherlands might be explained by a non- exhaustivity of the diagnoses in the years of birth of many patients. The wide clinical spectrum of NP-C was not recognized until the early 1990s, especially regarding rapidly fatal infantile cases, and no specific laboratory testing was available until the mid 1980s. For this review, an updated incidence of 0.82/100,000 was calculated for France, considering the total number of cases (n = 63) diagnosed for French hospitals during the 2000-2009 period *vs*. the number of births during the same period, a possibly more appropriate way of calculation. This value should be considered as a minimal estimate, since atypical phenotypes may not be suspected clinically or may be missed by the diagnostic laboratory. Including prenatal cases from terminated pregnancies during the same period (n = 11) increased the incidence to 0.96 per 100,000.

Most families (about 95%) belong to the NP-C1 group. Two NP-C1 isolates have been described. The first one, in French Acadians originating from Normandy and originally established in Nova Scotia, was initially described as Niemann-Pick disease type D; it is characterized by the *NPC1 *p.G992W mutation [1, 17, and 24]. Another isolate was described in Hispanics from southern Colorado and New Mexico with their roots in the Upper Rio Grande valley of the USA, carrying the *NPC1 *p. I1061T mutation [25,26].

## Clinical Description

The clinical presentation of NP-C is extremely heterogeneous, with an age of onset ranging from the perinatal period until well into adult age (as late as the seventh decade of life). Similarly, the lifespan of the patients varies between a few days until over 60 years of age, although a majority of cases die between 10 and 25 years of age [10,11,27-30]. The clinical spectrum discussed below has been analyzed from several large surveys [28-35].

NP-C is classically a neurovisceral condition. Importantly, visceral involvement (of liver, spleen, and sometimes lung) and neurologic or psychiatric manifestations arise at different times, and they also follow completely independent courses. Apart from a small subset of patients who die at birth, or in the first 6 months of life from hepatic or respiratory failure, and exceptional adult cases, all patients ultimately will develop a progressive and fatal neurological disease. Systemic disease, when present, always precedes onset of neurological symptoms, but the systemic component may be absent or minimal in approximately 15% of all patients, and close to half of the adult-onset patients, at least at the time of diagnosis. In typical patients, the neurologic disorder consists mainly of cerebellar ataxia, dysarthria, dysphagia, and progressive dementia, and the majority of cases show a characteristic vertical supranuclear gaze palsy (VSGP) [36]. Cataplexy, seizures, and dystonia are other quite common features, and psychiatric disturbances are frequent in late-onset patients. The proper recognition of VSGP is essential but this sign is often overlooked at an early stage, because slow pursuit is often maintained although saccade velocity is already impaired. Cataplexy (with or without narcolepsy), usually laughter-induced, is another more specific symptom [37,38]. Except for the perinatal period, the systemic disease is usually not very severe and is well tolerated. The splenomegaly has been described to fluctuate and to decrease with time. Severe lung involvement has been reported in a few patients but is not frequent.

**Figure 1:**
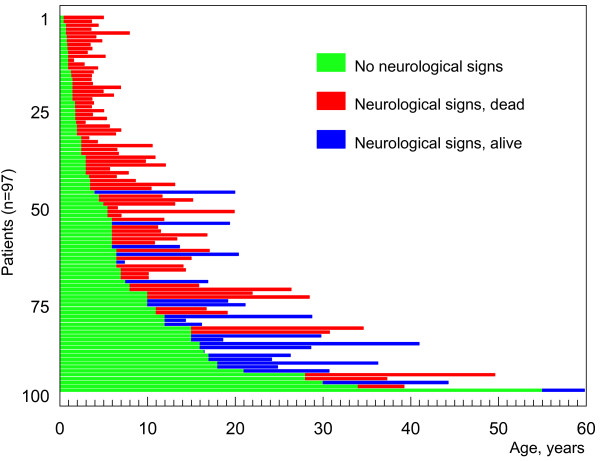
**Age of onset of neurological disease vs lifespan**. Study on 97 cases for whom appropriate clinical information was available from the cohort of 181 patients originating from French hospitals. Each horizontal bar depicts one patient. The green color indicates the period during which the patient did not present neurological symptoms, irrespective of the presence or absence of preexisting systemic disease. Patients who died in their first days or months of life from systemic disease (n = 19) are not shown on this graph.

**Figure 2:**
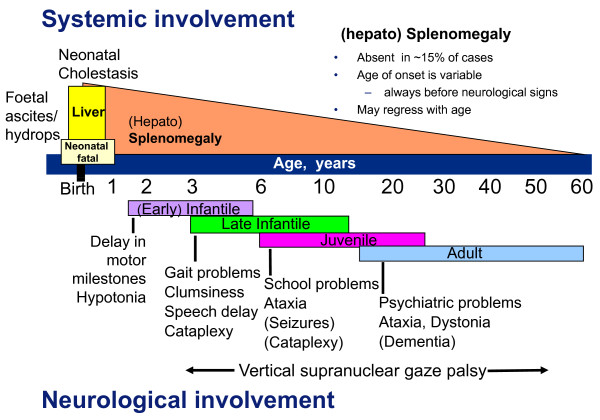
**Niemann-Pick disease type C as a neurovisceral disease**. Schematic representation of the main forms of the disease, with particular emphasis on type and age of onset of first neurological symptoms

A description of the various clinical forms by age categories has been used in recent reviews [10,11,27] and will also be followed in this summary. Detailed complementary information can be obtained in [27]. For each age category except for the perinatal presentations, one should however distinguish patients entering the disease by systemic involvement [39] from those who are starting then their neurological disease (although they may have presented earlier with visceral symptoms). Of essential importance is to note that the age of onset of the systemic symptoms is not related with that of the neurological disease (the latter can occur many years or even decades later), while there is a correlation between the age of onset of the neurological symptoms and the general further course of the disease and lifespan (Fig. 1). Categorizing patients by forms based on the age range of onset of the neurological symptoms`[11,32,40], irrespective of the age of the first symptom, is very useful for genetic counseling, natural history studies and also in clinical practice. With an exception for the severe early infantile neurological form which is quite significantly distinct, recent large studies have however demonstrated an overlap between the neurological forms, and thus a continuum [27]. A schematic representation is proposed in Fig. 2.

### Perinatal presentation

Niemann-Pick C disease is now recognized as a relatively common cause of liver disease in early life. Fetal hydrops or fetal ascites can be observed [28]. Above all, a prolonged neonatal cholestatic icterus, appearing in the first days or weeks of life and usually associated with progressive hepatosplenomegaly is present in close to half of patients, although with very variable intensity [29,41,42]. In most cases, the icterus resolves spontaneously by 2 to 4 months of age, and only hepatosplenomegaly remains for a highly variable period, preceding onset of neurologic symptoms (see below). In about 10% of these patients, however, the icterus quickly worsens and leads to liver failure. Children with this dramatic "acute" neonatal cholestatic rapidly fatal form usually die before the age of 6 months [29]. Some other infants, especially (but not exclusively) those having mutations in the *NPC2 *gene, present with a severe respiratory insufficiency (together with hepatosplenomegaly or more severe liver disease) that may also be fatal. In two patients, lung lavage, radiology and histology showed signs of pulmonary alveolar lipoproteinosis [43,44]. Patients with NP-C do not show neurological manifestations during the neonatal period (important for differential diagnosis). But there are many examples of patients dying from a severe perinatal form having siblings with a neurologic infantile or juvenile onset form [11,29].

### Early infantile period (2 months-2 years)

#### Systemic stage

An isolated hepatosplenomegaly can be discovered at this age period, which may well stay isolated for many years, in spite of the early onset. Once the diagnosis of NP-C is made, a regular neuropediatric follow up should be initiated.

#### Severe early infantile neurologic onset form

In these infants, hepatosplenomegaly has almost invariably been present since birth or the first months of life. Delay of developmental motor milestones from the age of 8-9 months and central hypotonia constitute the first neurologic symptoms, which become evident between the age of 1 and 2 years. Subsequent clinical course includes a loss of acquired motor skills, proportionally less marked mental regression, followed by pronounced spasticity with pyramidal tract involvement. Many of these children never learn to walk. Intention tremor is frequently present; supranuclear gaze palsy is usually not recognized. Seizures are uncommon. Brain imaging (MRI and MRS) shows signs of leukodystrophy and cerebral atrophy. Survival rarely exceeds 5 years. This form seems to be more frequent in Southern Europe (where it constitutes *>*20% of the cases) and the Middle East [11,29,32].

### Late infantile period (2 to 6 years)

#### Systemic stage

Many patients start their disease by discovery of an isolated hepatosplenomegaly or splenomegaly during this period. Regular neuropediatric follow-up should be initiated, as above.

#### Late infantile neurologic onset form

Hepatosplenomegaly has almost invariably been present for a varying length of time. Language delay is frequent. The child often presents with gait problems, frequent falls and clumsiness between 3 and 5 years of age, due to ataxia. VSGP is usually present but may not be recognized at an early stage. Hearing loss has also been described. Cataplexy develops relatively frequently and may occasionally be the presenting symptom. The motor problems worsen, and impairment in mental development becomes more obvious. A significant proportion of patients develop seizures which may be partial, generalized, or both. They generally respond to standard treatment but refractory cases may occur, with some patients dying from status epilepticus or complications of seizures. Severe epilepsy has a bad prognosis and significantly shortens the lifespan of the patients. As ataxia progresses, dysphagia, dysarthria, and dementia develop. At later stages, the patients develop pyramidal signs and spasticity, and pronounced swallowing problems. Most require gastrostomy. Death most often occurs between 7 to 12 years in this form.

### Juvenile period (6-15 years) (classical form)

#### Systemic stage

Discovery of an isolated splenomegaly (or, rarely, of a hepatosplenomegaly) at this period may again be the inaugural sign of the disease, and these patients should later be appropriately monitored.

#### Juvenile neurologic onset form

This constitutes in most countries the most common form of the disease. A moderate splenomegaly (or hepatosplenomegaly) is frequent, and may have been detected at any earlier time, including the neonatal period. However, cases in whom a splenomegaly had been noted in early childhood but is hardly detectable at the time first neurological symptoms arise are not rare; and absence of a detectable organomegaly has been reported to occur in at least 10% of cases. School problems with difficulties in writing and impaired attention are very common and may lead to misdiagnosis. The disease may also mimic dyspraxia. VSGP is almost invariably present and often the initial sign. The child becomes clumsier and shows more learning disabilities. Cataplexy, with or without narcolepsy, typically laughter-induced, is another common symptom. Ataxia soon becomes obvious, with frequent falls and difficulties to run, and progresses at a variable rate. Dysarthria develops, as well as dysphagia. Action dystonia is also frequent, Motor impairment is major and intellectual decline may be variable. About half of the patients with the classic form develop seizures of variable type and severity (see above). At a later stage, dysarthria worsens and patients often stop talking. At a late stage, patients develop pyramidal signs and spasticity, and pronounced swallowing problems, requiring gastrostomy. The lifespan is quite variable, some patients being still alive by age 30 or later [27].

### Adolescent and adults (>15 years)

#### Systemic adult form of NP-C

The finding of three patients aged 53-63 years with isolated splenomegaly and a biochemical and molecular diagnosis of NP-C [45-48] suggests the existence of a rare non-neuronopathic form of the disease (possibly corresponding to the ill-described historical "type E"). Nevertheless, apart from these exceptional cases and from infants with early death, as stated above, all NP-C patients develop neurologic symptoms.

#### Adult neurologic onset form

More patients with a neurologic adult onset form of the disease (often in the second or third decade, but as late as 50 years or older) have been described in recent years [30,35,49-53] This diagnosis is probably underestimated. Absence of clinically detectable splenomegaly has been reported in a significant proportion of patients but abdominal sonography may reveal a slightly enlarged spleen. VSGP is usually present but may also be missing. The most common symptomatology is that of an attenuated juvenile form with an insidious onset, with in at least one third of cases, a psychiatric presentation that may be isolated for several years before the onset of motor and cognitive signs. Psychiatric signs are most often consistent with psychosis, including paranoid delusions, auditory or visual hallucinations, and interpretative thoughts. Onset may be acute or progressive, eventually with relapses. At this stage the neurologic examination may be normal. Other types of psychiatric disturbances are depressive syndrome, behavioral problems with aggressiveness, or social isolation. Cases have also been reported with bipolar disorders, obsessive-compulsive disorders, or transient visual hallucinations. From compilation of the literature [35] the most common features are: cerebellar ataxia (76%), vertical supranuclear ophthalmoplegia (75%), dysarthria (63%), cognitive troubles (61%), movement disorders (58%), splenomegaly (54%), psychiatric disorders (45%) and dysphagia (37%). Movement disorders (dystonia, Parkinsonism, chorea) are more frequent than in the juvenile form. Some patients show severe ataxia, dystonia, and dysarthria with variable cognitive dysfunction, whereas psychiatric symptoms and dementia dominate in others. Epilepsy is rare in adult onset NP-C (15%). Later course is similar to that in the juvenile form.

## Etiology

Mutations in either of the two genes, *NPC1 *or *NPC2*, may cause the disease [13-16]. Approximately 95% of patients have mutations in the *NPC1 *gene, which encodes a large membrane glycoprotein with mostly a late endosomal localization [54]. The remainder have mutations in the *NPC2 *gene, which encodes a small soluble lysosomal protein that binds cholesterol with high affinity [16,55,56]. Mutations in the *NPC1 *or *NPC2 *genes result in a similar cellular lesion, including a unique impairment in processing and utilization of endocytosed cholesterol that could explain cholesterol storage and secondary alterations of sphingomyelin metabolism in extra neural tissues. Glycolipids and free sphingosine/sphinganine storage also occurs. In brain, - more specifically in neurons - the dominant lipid accumulation is in fact that of GM2 and GM3 gangliosides, with only limited apparent abnormalities of cholesterol (see below). Early studies in cells and tissues from NP-C1 and NP-C2 patients could not disclose any biochemical marker that was specific to any of the groups, suggesting that both proteins may function in tandem or sequentially [14]. Comparison between double mutant mice deficient in both NPC1 and NPC2 and the single mutants demonstrated a non-redundant functional cooperativity of the two proteins in a common pathway for lipid cellular transport, which strengthened this concept [18]. The exact functions of the NPC1 and NPC2 proteins are still unclear [10-12,56,57], which greatly complicates understanding of the pathophysiology. Neuronal storage with meganeurite formation and extensive growth of ectopic dendrites, as well as formation of neurofibrillary tangles, are important neuropathological features together with neuroinflammation and neuroaxonal dystrophy. As the disease progresses, neuronal death becomes prominent, affecting more specifically certain regions, particularly Purkinje cells of the cerebellum, but the basis of this selective neuronal vulnerability is still unknown [10,58].

### Lipid accumulation in tissues

Similar profiles have been observed in NP-C1 and NP-C2 patients (and animal models), but the pattern of accumulating lipids is different in brain and in non-neural organs [10,18,20,40,59-64]. In liver and spleen, a complex pattern, with no predominating compound, is observed. Accumulated lipids include unesterified cholesterol and sphingomyelin (2- to 5-fold increase in human patients), bis(monoacylglycerol) phosphate (also named LBPA or BMP), glycolipids (essentially glucosylceramide and lactosylceramide), and free sphingosine and sphinganine. In human patients, the level of storage is more pronounced in the spleen than in the liver, where changes may be subtle. In brain tissue, neither cholesterol nor sphingomyelin overtly accumulate, but significant alterations of glycosphingolipids occur, especially for gangliosides GM2 and GM3 (10-20 fold increase). Free sphingosine levels are much less elevated in brain (x3) than in liver or spleen (x20) [62,64]. Myelin lipids are markedly affected in the NPC1 mouse model but in patients, a significant decrease is only seen in the early infantile neurological onset form [18,60].

### Cell biology and cholesterol transport, and the brain enigma

Initial studies by Peter Pentchev and associates and further work from several laboratories (reviewed in [10,20]) demonstrated, in cultured skin fibroblasts of Niemann-Pick C disease patients, a disruption in intracellular transport of endocytosed cholesterol. Endocytosed low density lipoproteins are delivered to late endosomes/lysosomes, where they are hydrolyzed, so that free cholesterol is released. In normal cells, this cholesterol is transported rapidly out of endosomes to the plasma membrane and the endoplasmic reticulum. In Niemann-Pick C disease cells (either NPC1 or NPC2), the cholesterol does not exit the endocytic pathway but accumulates within lysosomes. This anomaly constitutes the cellular hallmark of the disease. Due to this sequestration, the subsequent induction of all low-density lipoprotein cholesterol-mediated homeostatic responses (more specially cholesteryl ester formation) is retarded in Niemann-Pick C disease cells. Normal responses can be induced by membrane-permeable oxysterol and by mevalonate, showing that the ability of the cell to respond is maintained. Very recently, it was further shown that the block in cholesterol delivery to the ER can also be overcome by 2-hydroxypropyl-beta-cyclodextrin [65], and that this compound added to fibroblasts reduces the lysosomal cholesterol accumulation [66]. Studies in patients' cells showed that lysosomal storage of unesterified cholesterol may show a variable intensity, and a "variant" biochemical phenotype with mild abnormalities has been described [67,68]. Later work showed that this phenotype was underlined by specific *NPC1 *mutations (see below). Unexpectedly, fibroblasts from a large proportion of obligate heterozygotes have been found to show mild but definite changes [67-70].

This unique impairment in processing and utilization of endocytosed cholesterol obviously plays a key role in the pathogenesis of Niemann-Pick C disease, and, at least in extraneural organs, could actually explain a more general dysfunction of intracellular metabolism of lipids [63]. Sphingomyelin accumulation appears related to lysosomal cholesterol storage, since sphingomyelinase activity can be strongly modulated in fibroblast cultures of Niemann-Pick C disease patients by incubation in presence or absence of low-density lipoprotein. Cholesterol accumulation might also modulate glucosylceramide hydrolysis [71], as well as the trafficking of late endosomal proteins such as Rab 9 and mannose-6-phosphate receptors [72], two key players in the normal function of the endosomal/lysosomal system. There is thus good evidence that cholesterol accumulation in the late endosomal/lysosomal compartment can impair vesicular trafficking pathways.

The pathogenesis of the neuronal dysfunction appears by far more complex, since brain cholesterol is synthesized locally, mostly by oligodendroglial cells and to a lesser extent by astrocytes and neurons. Neurons might also acquire a small amount of cholesterol by glial delivery through apo-E uptake [73]. By chemical measurement, no significant increase of cholesterol concentrations can be found in dissected cerebral grey matter from human patients [60]. *In situ *labeling using filipin histochemistry, however, reveals a sequestration of unesterified cholesterol in cell bodies of neurons and glia of single NPC1 or NPC2 mutant mice as well as those of the double mutant [18,58,73-75]. These observations are not necessarily contradictory, since studies on cultured sympathetic neurons from NPC1 mutant mice gave indication that cholesterol did accumulate in cell bodies, but was decreased in distal axons, leading to a distribution imbalance [76,77]. One group has reported that endogenously synthesized cholesterol may significantly contribute to the overall cholesterol accumulation observed in Niemann-Pick C disease in various cell types, including glial cells [78]. Nevertheless, since abnormal filipin staining of neurons is also observed in a wide spectrum of other lysosomal storage disorders [discussed in 63], the exact participation of cellular cholesterol transport abnormalities in the pathophysiology of the neurodegenerative NP-C disease remains elusive. Of note, fibroblasts from patients with an adult-onset of neurological symptoms may show either a severe cholesterol trafficking defect or only minimal alterations (biochemical variant) [35,69,70]. Conversely, in two "variant" siblings who had died from a juvenile form, the liver showed no lipid accumulation (spleen did), but the brain showed typical accumulation of GM2 and GM3 gangliosides [79].

### The NPC1 and NPC2 proteins

The mature native NPC1 is a large (1252 amino acids) glycoprotein with 13 transmembrane domains, that resides primarily in late endosomes and interacts transiently with lysosomes and the trans-Golgi network [54,80]. It possesses a sterol-sensing domain (amino acid residues 615-797) showing homologies with those of HMG-CoA reductase, SCAP, patched and NPC1L, the exact role of which is still unclear although it appears necessary for protein function. Two luminal domains may play a role in protein-protein interactions: a cysteine-rich loop with a ring-finger motif which harbors about 1/3 of the mutations described in patients (amino acid residues 855-1098), and a highly conserved domain with a leucine-zipper motif, located in the N-terminal tail (amino acids 25-264) [81]. Importantly, the latter has been shown to possess a cholesterol-binding site (reviewed in [56]). Contrary to the NPC1 protein, the NPC2 protein is small (132 amino acids), soluble, secreted and recaptured. It is transported to the lysosome via the mannose-6-phosphate receptor and binds cholesterol with submicromolar affinity [56]. The mutation p.S120P (observed in a patient with a juvenile neurological onset and slowly progressive form [82]) has been instrumental to confirm the functional significance of the cholesterol-binding site of the NPC2 protein [83]. Studies in patients and animal models have shown that both NPC2 and NPC1 are required for cholesterol egress from the lysosome. Binding of cholesterol to NPC1 and dissociation both appear accelerated by NPC2 [83]. Based on the current stage of knowledge but fully compatible with earlier studies (reviewed in [56]) a "handoff" model has recently been proposed for the coordinated role of the two proteins [19]. In this model, cholesterol released within the lysosome binds to NPC2 with its hydroxyl group exposed; a transfer to the N-terminal domain of NPC1 occurs reversing its orientation, so that the hydrophobic side chain could lead the way into the membrane and/or the glycocalix. The most recent studies [65] indicate that the role of NPC2/NPC1 proteins in cholesterol transport is restricted to lysosomal export. Current data suggest that retrograde cholesterol movement from the plasma membrane to the ER does not require NPC1 [65] and implication of these proteins in cell processing of endogenously synthesized cholesterol [84] is still a matter of discussion.

Many uncertainties thus remain regarding the precise and complete functions of the NPC1 and NPC2 proteins. It has also been suggested that they could be involved in fusion/fission events between the late endosome and the lysosome. One important (and yet unanswered) question is whether these proteins - at least NPC1 - also directly regulate or mediate retrograde transport of other lysosomal cargo. Glycolipids, which constitute the main lipid accumulation in the brain, by opposition to the quantitatively minor cholesterol imbalance in neurons, are good candidates. The storage of GM2 and GM3 gangliosides in brain is not specific. Yet, the increase of GM2 occurs much earlier and is more prominent in NP-C than in other lysosomal diseases [63]. But no data supportive of a glycolipid transport by NPC1 or NPC2 have been published so far. It has also been postulated that sphingosine storage could be the primary trigger of a pathogenic cascade in NP-C since this lipid can disrupt calcium homeostasis in NPC1 lysosomes [85,86]. The latter studies, however, were conducted in non neural NP-C cells or in a drug (U18666A)-induced model. In brain, currently available data show a close link between accumulation of the different lipids, both in developmental terms and after therapeutic attempts [63,64]. No ganglioside or sphingosine accumulation can be detected in the brain of human fetuses at 24 gestational weeks, although the liver already shows a pronounced storage. Arguments for and against each of the accumulated lipids as the offending metabolite have recently been discussed [86]. Most likely, stored lipids (and possibly other metabolites) collectively contribute to the pathology. More work is clearly needed to better understand the cause of brain dysfunction in Niemann-Pick C disease. In particular, the mechanisms by which Purkinje cells and other neurons degenerate remain unclear.

### Disease-causing mutations and genotype-phenotype relationships

The Niemann-Pick type C disease variation database [87] listed by January 2010 244 *NPC1 *and 18 *NPC2 *gene sequence variants. Reporting from diagnostic laboratories, however, has not been exhaustive, and the current number for identified *NPC1 *disease-causing mutations is most likely close to 300. More than 60 polymorphisms of *NPC1 *have also been described, some of them very common. In early genetic complementation studies, it was stated that about 95% of the families had mutations in the *NPC1 *gene [14]. In France, among the 132 families genotyped so far, 9 had *NPC2 *mutations. To date, only c:a 30 families have been identified worldwide with mutations in the *NPC2 *gene. Several large mutational studies have been published [33,47,82,87-97], but only few functional studies [47,82,91,98-101].

The *NPC1 *gene, mapped to chromosome 18q11-q12, spans 56 kb and contains 25 exons. One mutant allele, p.I1061T, is particularly frequent [26] (approximately 20-25% of alleles in patients diagnosed in France or the United Kingdom). This mutation is also highly prevalent in patients from a Spanish-American isolate from the upper Rio Grande valley, but much less frequent in Portugal, Spain or Italy [91,94,96]. In the homoallelic state, it is associated with prominent cellular cholesterol trafficking abnormalities in fibroblasts of patients, and it correlates with a juvenile neurologic onset form of the disease [26]. In the heteroallelic state, so far it has never been found associated with the most severe infantile neurologic onset form. The I1061T mutant was shown to be a functional protein selected for endoplasmic reticulum-associated degradation due to protein misfolding and thus a potential target for chaperone therapy [98]. The second most recurrent *NPC1 *mutation in Europe, p.P1007A, is the prototype of a "biochemical variant" mutation [47,89,95]. In the homozygous state, it has been described in a family with two adult onset siblings [91]. A number of other recurrent *NPC1 *mutations seem to be associated with adult neurological onset of the disease [35,95]. The mutation p.G992W, typical of Nova-Scotian patients [17] is sporadically (but rarely) found in patients of other origin. As more patients are genotyped, a larger number of recurrent mutations are observed, some of them preferentially found in patients from defined ethnic origin.

The few genotype-phenotype studies published so far in NP-C1 patients generally showed good correlation between nonsense or frameshift mutations and the most severe neurologic course. Missense mutations have emphasized the functional significance of two particular domains of the NPC1 protein. Homozygous mutations in the sterol-sensing domain were found to be very deleterious, corresponding to a lack of mature NPC1 protein and to a very severe disease phenotype, biochemically and clinically [47]. The cysteine-rich luminal loop contains approximately one third of all described mutations, with a variable cellular and clinical impact [47,89,93,95]. Among others, it harbors the three most frequent mutations discussed above. Interestingly, mutations leading to a less severe impairment of cellular trafficking ("variant" phenotype) are typically located on this loop [47,90,91,93-95]. Genotype-phenotype correlations for more specific mutations have been discussed in earlier reports [11,88,95].

The *NPC2 *gene (initially known as *HE1*), mapped to chromosome 14q24.3, spans 13.5 Kb and contains 5 exons [16]. One nonsense mutation (E20X) appears relatively frequent [82,92], and many other mutations also lead to a truncated protein. They have so far been associated with very severe clinical phenotypes. Described missense mutations have corresponded to more varied phenotypes, including juvenile and adult onset patients [82,92,93,101].

Finally, for both *NPC1 *and *NPC2*, the study of a large number of multiplex families has clearly shown that mutations correlate with the neurological form of the disease, but not with the systemic manifestations [11].

## Diagnostic methods

**Figure 3:**
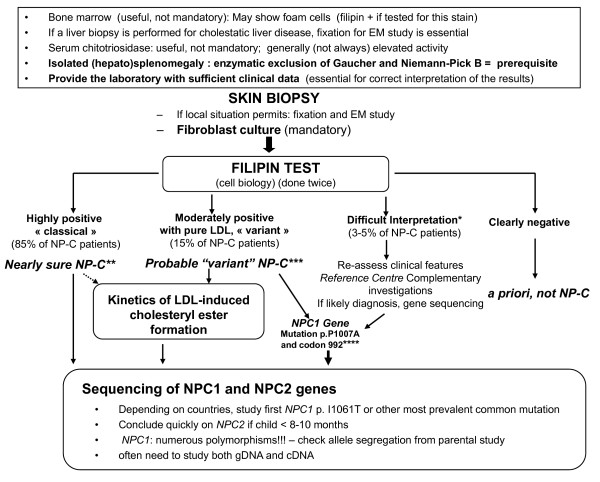
**Laboratory diagnosis algorithm**. Footnote: This algorithm is as proposed in Wraith et al. *Mol Genet Metab *2009, **98**:152-165 [27] *Sphingomyelinase deficiency (including late-onset type A) may give a dubious filipin pattern, with normal kinetics of LDL-induced cholesteryl ester formation ** False positive: I-cell disease (but very different clinical features) ***Heterozygotes may show a pattern (filipin staining and kinetics of LDL-induced cholesteryl ester formation) similar to that in "variant" patients ****In many countries, *NPC1 *p.P1007A or different missense mutations on codon 992 constitute the most frequent "variant" mutations Genetic studies can also be undertaken if clinical symptoms are very suggestive of a diagnosis of NP-C, even with negative results from filipin testing.

The laboratory diagnostic algorithm proposed in a recent consensus report [27] is given in Fig. 3.

### Initial clinical assessment

**Figure 4:**
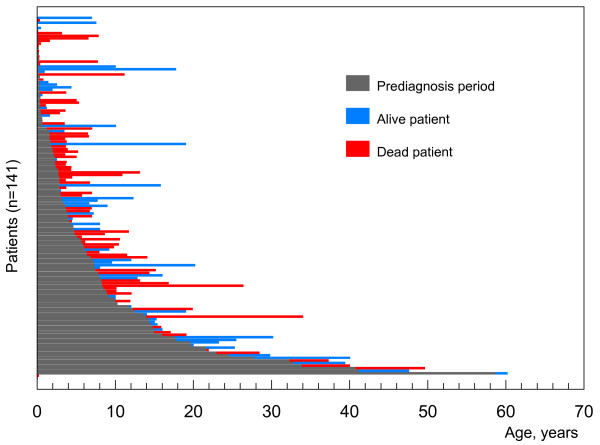
**Age at diagnosis vs life span**. Study in a cohort of 141 patients originating from French hospitals. Each horizontal bar depicts one patient. The change of color shows at which age the diagnosis was established. Median: 3.1 years; 0-9 months: 30%; < 2 years: 36%; < 5 years: 60%; > 16 years: 12%

Suspecting Niemann-Pick disease type C is relatively easy in patients with the most typical symptoms, such as combined splenomegaly, ataxia, and supranuclear vertical gaze palsy. However, as described earlier, strikingly different clinical presentations exist, especially in infants and neonates. The fact that isolated spleno- or hepatosplenomegaly can be the presenting symptom long before neurologic onset has not been emphasized enough. Finally, the diagnosis is often very delayed (and probably often not made) in neurological cases lacking organomegaly, and in psychiatric cases. Consequently, the age at which the diagnosis is established is very variable. This is illustrated by data obtained in the author's laboratory for a representative cohort of patients (Fig. 4).

The characteristic key signs and symptoms in the systemic, ophthalmological and neuropsychiatric areas have been discussed above and the reader is also referred to a recent review [27]. A comprehensive clinical examination should be performed. The neurological evaluation must include muscle tone and strength tests, motor reflexes, assessment of movement (ataxia and dystonia) and swallowing testing [27]. Psychometric assessment is also important.

The ophthalmological assessment is of particular importance, because abnormal saccadic eye movements (SEM) are often the earliest neurological sign in NP-C. Proper examination is not always done, and reported findings are sometimes neglected in the global evaluation of the patient. The initial SEM deficit is in the vertical plane (downward, upward, or both). VSGP can be described as an increased latency of initiation of vertical saccades, with gradual slowing and eventual loss of saccadic velocity [27,102,103]. Subsequently, horizontal gaze is also affected. Cataplexy ranges from subtle signs (minor head-drop or falls, often confused with seizures) to full collapse in response to humorous stimuli [27].

### Neurophysiologic and neuroradiologic studies

Hearing tests (audiogram and/or brainstem evoked potentials) often show abnormalities. MRI and CT scans are not very useful for diagnosis, as they may be normal or show cerebellar or cortical atrophy, or, in the severe infantile form, white matter changes. Some rare patients have been described with a peripheral neuropathy.

### Histology

Foam cells and sea-blue histiocytes are usually - but not always - present in bone marrow. Foam cells stain strongly positive with filipin. Ultrastructural studies on skin [104], conjunctival, or liver biopsies can provide strong support for the diagnosis, but false-negative results often occur on liver biopsy studied by light microscopy only [41].

### Non-specific laboratory analyses

Routine laboratory tests usually give normal results, except in patients with cholestatic jaundice or hypersplenism. Low HDL-cholesterol is a frequent but not universal finding. Plasma lipid profiles seem correlated to severity of cholesterol trafficking abnormalities [97]. Chitotriosidase activity is usually mildly elevated [105] but can be normal. Acid sphingomyelinase activity is normal or elevated in leukocytes (differential diagnosis with Niemann-Pick type B or atypical type A) but often partially deficient in fibroblasts [11,25,29,67].

### Specific laboratory diagnosis

#### Biochemical/cell biology study: the "filipin" test

The demonstration of impaired intracellular cholesterol transport and homeostasis is considered the primary diagnostic test for NP-C. These studies require living cells and thus a skin fibroblast culture. They should be conducted in specialized centres with the required experience. The "filipin test" is the most sensitive and specific assay. Fibroblasts are cultured in a LDL-enriched medium, then fixed and stained with filipin (a compound forming specific complexes with unesterified cholesterol). Fluorescence microscopic examination of NP-C-positive cells typically reveals numerous strongly fluorescent (cholesterol-filled) perinuclear vesicles. This "classical" storage pattern is observed in approximately 80-85% of cases. A lesser (and variable) level of storage is seen even under optimized conditions [67] in the remaining cases, described as having a "variant" biochemical phenotype [67,68]. As discussed above, several recurrent NPC1 mutations are known to result in this "variant" biochemical phenotype. Note that a similar, mildly abnormal filipin pattern, has been observed in a number of heterozygotes [69,70], but also not infrequently in acid sphingomyelinase deficiencies. Measurement of the LDL-induced rate of cholesteryl ester formation was until recently systematically used as a secondary test, showing very low levels in cell lines with a "classical" biochemical phenotype but only a mild or non-significant impairment in those with a "variant" phenotype [67,68]. As this test is complex, costly and time-consuming, mutation analysis is now often initiated directly when the filipin study is clearly positive. From the experience of the author, based on the study of cells from more than 600 NP-C patients, demonstration of cholesterol accumulation in cultured fibroblasts provides a clear-cut diagnosis in a majority of cases, but making a decision can be very difficult in some cell lines showing only minor abnormalities. In such cases, (and eventually in cases with apparently negative filipin but a history highly suggestive of NP-C), complementary mutation analysis is very useful to reach a definitive diagnosis.

#### Genetic testing

It is highly advisable to undertake gene testing in every newly diagnosed patient, since molecular genetic study is today the highly preferred strategy for prenatal diagnosis, and the only reliable one for identification of carriers in blood relatives. Furthermore, as discussed above, gene testing can sometimes be necessary to confirm or disprove the diagnosis of NP-C. Genetic complementation studies - performed earlier in a few laboratories to define which gene was affected - are no longer used today, because cell hybridization and further testing are more elaborate than gene sequencing. Sequencing of all exons and boundaries is more laborious for the *NPC1 *gene (25 exons) than for the *NPC2 *gene (5 short exons), which is unfortunate, since over 95% of NP-C patients have pathological *NPC1 *mutations. Rapid methods have been published to test for the two most frequent mutations [26,47]. Identification of *NPC1 *mutations can, in some instances, be difficult and may require combined studies of gDNA and cDNA. All groups have met a common problem, namely that in some patients mutations could be identified in only one allele, and in a few of them, no mutation at all. The latter patients have raised the question of a potential third gene causing NP-C. This cannot be excluded, but often the possibilities of large deletions, or of deep intronic mutations [106] have not been investigated. Finally, due to the highly polymorphic nature of *NPC1*, interpretation of new missense mutations should be undertaken with caution.

## Differential diagnosis

In the neonate and young infant, Niemann-Pick disease type C must be differentiated from idiopathic neonatal hepatitis, and other causes of cholestatic icterus. Onset of cholestasis usually occurs in the early neonatal period. Associated splenomegaly is a useful orientation sign. In case of isolated splenomegaly or hepatosplenomegaly, NP-C should be considered as a possible cause. Among other lipidoses, the most obvious differential diagnoses are Niemann-Pick type B (similar foam cells in bone marrow) and Gaucher disease. In older children and adults, depending on the symptoms, other conditions with cerebellar ataxia, dystonia, cataplexy and supranuclear gaze palsy need to be considered [27,31].

## Genetic counseling

Niemann-Pick C disease is genetically inherited following an autosomal recessive mode. The genetic status of a blood relative can be reliably established if mutations have been identified in the family index case. However, it is not currently possible to ascertain the status of a person from the general population, due to the complexity of *NPC1 *gene sequencing and its polymorphic nature. Antenatal diagnosis is possible under the conditions described below.

The possibility of symptomatic heterozygotes has been raised in three families known to the author but ruled out in two of them (no further study in the third one). Two disease-causing *NPC1 *mutations had been identified in each index case. In both families, the father of the proband developed progressive symptoms compatible with an adult onset neurologic form of NP-C. Subsequent complete gene sequencing revealed one allele carrying the mutation transmitted to the affected child, and another (not transmitted) disease-causing mutation on the other allele (M.T. Vanier and K. Harzer; M.T. Vanier and A. Ivanoiu, unpublished). These individuals were thus NP-C1 homozygotes with an adult onset form. These exceptional histories illustrate some of the problems eventually posed by the clinical heterogeneity of NP-C and the possible underestimation of adult-onset form of the disease.

## Antenatal diagnosis

Prenatal diagnosis of NP-C should be offered to couples at risk [27,107,108]. It is best achieved using chorionic villus sampling (CVS) at 10-12 weeks, but is also possible on amniotic cells. Molecular genetic analysis is today by far the preferred strategy [27], for several reasons. Unlike the cellular biology testing using filipin staining, it does not require cultured cells and a lengthy elaborate work up. The results can be obtained much earlier in pregnancy, and the tests can in principle be set up in any good molecular biology laboratory. It however requires that mutations have been identified on both alleles in the index case, or at least that suitable intragenic markers have been identified in the nuclear family. Today, few laboratories offer a prenatal test using the cellular biology strategy, which should be considered as a last resort due to its many drawbacks. Results will not be reached until 5-7 weeks after the sampling; the tests are technically difficult; besides, they are fully reliable only when the proband has shown severe abnormalities, thus excluding 15-20% of the families.

## Management including treatment

To date, management remains largely symptomatic. Information and support to families can be obtained through organizations specifically devoted to Niemann-Pick diseases (in the United States, United Kingdom, Germany, Spain, Italy, Argentina, Australia, Poland), to lysosomal diseases (France) or to inherited metabolic diseases (The Netherlands) [see appendix for websites]. Genetic counseling should be made available for family members. For detailed guidelines on current management of patients, the reader is referred to a recent publication compiled by an international working group [27]. A study on the cost of illness associated with NP-C in the UK has recently been published [109].

### Symptomatic management

Seizures generally respond at least partially to antiepileptic drugs until a fairly advanced stage of the disease. Cataplexy can usually be controlled by clomipramine, protriptyline, or modafinil. Anticholinergic agents have been reported to improve dystonia and tremor in some patients. Physiotherapy is useful in the management of spasticity and the prevention of contractures. Melatonin may be used to treat insomnia. Patients with a slow disease course may benefit from special schooling for handicapped children. Proper management of infections and of feeding difficulties (gastrostomy) is essential at an advanced stage of the disease.

### Specific treatment

In the murine and feline NPC1 models, bone marrow transplantation (BMT) did not improve the neurological disease, not unexpectedly considering the properties of the NPC1 protein; similarly, after BMT the neurologic status of a child continued to deteriorate, although there was a regression of hepatosplenomegaly and lung infiltration [110]. In addition, liver transplantation performed in a few cases with cirrhosis did not influence the course of neurologic deterioration [111]. On the contrary, because the NPC2 protein is soluble, secreted and recaptured, there is a rationale supporting early hematopoietic stem cell transplantation in NP-C2 patients [82]. The long-term outcome is yet unknown, but encouraging results have recently been obtained in one patient transplanted at 18 months and followed up until 3 years of age [112].

Treatment strategies based on the hypothesis that cholesterol is the offending metabolite were first proposed in the early 90's. The combination of hypocholesterolemic drugs and a low-cholesterol diet seemed to partially reduce the cholesterol load in liver, but no amelioration of the neurological disease was seen in patients after 2 years of treatment [31].

Since glycolipid storage appears to contribute to at least some of the neuropathologic features, an iminosugar inhibitor of glucosylceramide synthase (miglustat, also known as N-butyl-deoxynojirimycin, NB-DNJ and OGT 918, later approved for substrate reduction therapy of mild to moderate type 1 Gaucher disease), was administered to *npc1 *mutant mice and cats. It resulted in delayed onset of the neurological symptoms in both species, and a 20% longer survival of the mice [113]. A controlled clinical trial was thus initiated in neurologically symptomatic patients, first in adolescents and adults (12 years and above) [114], then in children (4-12 years). Long-term data from open-label extension treatment (up to 66 months) have now been reported in children [115] as well as in juvenile and adult patients [116] (reviewed in [27]). Overall, the disease course stabilized in 72% of patients treated for one year or more, based on a composite assessment of horizontal saccadic eye movement velocity, ambulation, swallowing and cognition. In January 2009, the European Union has extended the indication of miglustat to the treatment of progressive neurological manifestations in adult and pediatric patients with NP-C, and the drug is now approved for this indication in several other countries. This represents the first specific treatment for NP-C. Apart from single case reports [117,118], an international, multicenter observational cohort study in 66 patients treated off-label with miglustat has been published [119]. Evaluation made with a modified disease-specific disability scale [32] further showed a significant reduction in the annual rate of progression of the disease in a majority of patients. Late-onset forms generally appeared as the best responders. A further case series from Spain has been documented [120]. Longer term studies will be important to better evaluate the disease progression following the stabilisation phase [121]. Indication, clinical utility and monitoring of treatment with miglustat have been recently discussed [27,122]. In short, it has been recommended to treat patients as soon as they show neurological manifestations of any type. Due to the known adverse effects, such as diarrhea, flatulence, weight loss and tremor, it is not recommended today to treat patients with systemic disease only. Note that miglustat is not expected to have an effect on the systemic manifestations of NP-C.

### Disease monitoring

In order to monitor disease progression and, if applicable, patient responses to treatment, it is important to regularly quantify the degree of disability resulting from neurological impairment. Two disease-specific disability scales have recently been proposed [32,123]. The first one [32] (Table 1) evaluates four key parameters: ambulation, manipulation, language and swallowing, with a 4 to 5 point scale for each. This allows calculation of a composite score representing overall "functional disability". Although not formally validated, it has already been used successfully in several cohort studies. Recent natural history surveys using these different scales both concluded to a linear clinical progression over time [123,124]. The cohort including a broader - and thus more representative - range of clinical phenotypes [124] showed a more rapid course in the patients with an early onset.

**Table 1:** NP-C functional disability scale (from [32] and [27])

Ambulation	Score	Language	Score
	
Normal	1	Normal	1
Autonomous ataxic gait	2	Mild dysarthria^d^	2
Outdoor assisted ambulation	3	Severe dysarthria^e^	3
Indoor assisted ambulation	4	Non-verbal communication	4
Wheelchair bound	5	Absence of communication	5
			

**Manipulation**		**Swallowing**	
	
Normal	1	Normal	1
Slight dysmetria/dystonia^a^	2	Occasional dysphagia	2
Mild dysmetria/dystonia^b^	3	Daily dysphagia	3
Severe dysmetria/dystonia^c^	4	NG tube or gastric button feeding	4

Abbreviation: NG, nasogastric; ^a ^autonomous manipulation; ^b ^requires help for tasks but able to feed self; ^c ^requires help for all activities; ^d ^understandable; ^e ^only comprehensible to certain family members.

Useful monitoring tests have been recently discussed in detail [27]. Several methods for analysis of movement abnormalities [125,126] or neuropsychological profiles [127] have also been proposed. Results on three patients indicated that longitudinal MRS studies [128] might prove useful for follow up of therapy [129]. Diffusion tensor imaging has also been proposed [130].

## Experimental therapeutic approaches in animal models

Extensive research towards other therapeutic avenues is currently underway on animal and cellular models. These approaches have been reviewed in [27]. Various transgenic mice have been generated, such as mice over expressing Rab9, a protein involved in intracellular trafficking [131-133], or mice expressing NPC1 only in one particular brain cell type [134]. Most studies have however been conducted on the *npc1*^nih ^mouse and a cat model (both spontaneous *npc1 *mutants) [135,136], as well as a transgenic *npc2 *mouse mutant [18]. These animals are particularly useful to study brain dysfunction, and facilitate various types of experiments, including administration of various compounds with a therapeutic goal. Data have been published in the mouse using imatinib [137], curcumin [85], non-steroid anti-inflammatory drugs [138], neurosteroids (allopregnanolone) in combination with 2-HP-ß-cyclodextrin [139], and with 2-HP-ß-cyclodextrin alone [64,140]. Chronic subcutaneous administration of high doses of 2-HP-ß-cyclodextrin resulted in a striking reduction of the various stored lipids both in the liver and the brain of NP-C mice, as well as a very significant effect on their lifespan [64]. An orphan drug designation has been seeked for this compound from the US-FDA. However, translation of most of these studies to human patients is not straightforward. Even neglecting adverse effects [141] or the purity or homogeneity of certain compounds, a quite general and major limitation is the usual early timing of treatment (usually long before symptoms appear). Such experimental work in the whole animal is, however, important as it is generally felt that future treatment plans will combine several approaches and will be tailored to the individual.

## Prognosis

NP-C is a severe disorder that invariably leads to premature death, with few exceptions (three proven cases aged 53 years or more with isolated splenomegaly are known) [45-48]. However, as discussed above, the rate of progression and life span show considerable variation. The systemic disease can be fatal in early infancy. Patients with fetal hydrops survive at most a few days. Liver failure causes rapid death (before 3-6 months of age) in approximately 10% of neonates presenting with a cholestatic icterus, and a few patients (most of them with a severe *NPC2 *mutation) have died from severe pulmonary insufficiency. Neonatal cholestatic icterus is otherwise transient and usually resolves spontaneously by 4 months of age. Splenomegaly very rarely leads to hypersplenism. An important observation is that the age of onset of the systemic disease is generally unrelated to the subsequent neurological involvement and cannot be used as a predictor. This is well illustrated in Fig. 4, where several patients diagnosed in their first months of life are now teenagers. In the vast majority of patients, the lifespan is in large part determined by the age of onset of nervous system involvement. Data on large cohorts of patients recently compiled for Spain, the UK and France [27] are well in line with earlier reports. Patients with the severe neurologic early infantile form often die between 3 and 5 years of age, those with a late-infantile neurologic onset usually between 7 and 12. Patients with a juvenile neurologic onset survive until adolescence or later, with a sizable proportion reaching the age of 30. In a review of 68 cases with adult onset [35], the mean age at death (on 20 patients) was 38 ± 10.2 years, but some patients have reached the age of 70. Motor involvement is often more severe and more rapidly progressive than mental retardation. Progressive and severe dysphagia requiring gastrostomy is a common complication. Severe and intractable epilepsy accelerates the downhill course of the disease. Psychiatric disturbances, in rare cases, may be prominent or even dramatic.

Regarding recurrence within a sibship, the study of many multiplex families has shown that as a rule, the neurological form - as defined by age of onset of neurological symptoms, and irrespective of the age of onset of the systemic disease - is similar between siblings. The subsequent course can however show variations, especially for cases developing severe epilepsy. On the other hand, there are many examples of families with one case of fetal hydrops or fatal neonatal liver disease and a sibling having a more classical neurovisceral form - more often of the early infantile type, but also of the late infantile or juvenile type.

Correlations between the neurological form and the severity of the cholesterol trafficking lesion as found by the filipin test has been discussed previously [11,67,70]. In brief, in the experience of the author, a "variant" biochemical phenotype tends to correlate with a less rapid course, since it has so far never been found in the most severe early infantile neurological form, is rare in late infantile forms, but seen in a number of juvenile and nearly half of the adult onset patients. On the other hand, finding a very severe cholesterol trafficking impairment (massive cholesterol accumulation in lysosomes) is not predictive of any form of the disease (seen in the other half of adult-onset patients).

Finally, although genotype-phenotype correlations are limited, in NP-C1, some degree of prediction is often possible. Thus far, the p.I1061T allele has not been associated with the most severe infantile neurological form [11,47]. Frameshift or nonsense mutations, as expected, but also missense mutations affecting the sterol sensing domain usually have a severe impact. On the other hand, association with a mutation leading (when in the homozygous state) to an adult onset form usually results in a slowly progressive juvenile or early adult onset form [95].

## Unresolved questions

NP-C is a disease with many unresolved questions. To begin with, the precise and complete function(s) of the NPC1 and NPC2 proteins are still largely unknown. Only few studies on cholesterol transport and metabolism have addressed the brain, in spite of the fact that brain has a cholesterol metabolism that is different from that in cells from systemic organs [142]. The nature of the primary offending metabolite in brain is also unknown. For these reasons, meaningful high throughput drug screening strategies are difficult to set up.

A major practical problem is the current lack of a biochemical test with sufficient specificity to be used for screening - or even better, diagnosis - that could be carried out on a blood sample. Having to start from a skin biopsy excludes NP-C from all "metabolic screens" and significantly contributes to the delay in diagnosis. Importantly, a recent pilot study indicates that plasma of patients with NP-C show a specific oxysterol profile that could be used as a biomarker [143]. This observation may impact the future diagnostic strategy.

As regards therapy, because the NPC1 protein, unlike many other lysosomal proteins, is not secreted and recaptured, many therapeutic strategies that are currently holding promises for the future seem not easily applicable to NP-C, including cell and gene therapy. Another difficulty to treat the brain dysfunction is the unknown nature of the primary targets. Along this line, the potential mode of action of some experimental compounds (among which is ß-cyclodextrin) remains a puzzling question. Finally, the broad clinical spectrum, as well as the lack of good disease markers and clinical endpoints, makes evaluation of therapeutic trials particularly difficult.

## List of abbreviations

CT: computerized tomography; 2-HP-ß-cyclodextrin: 2-hydroxypropyl-ß-cyclodextrin; LDL: low-density lipoproteins; NP-C: Niemann-Pick type C; NP-C1: Niemann-Pick type C disease with mutations in the *NPC1 *gene; NP-C2: Niemann-Pick type C disease with mutations in the *NPC2 *gene; MRI: magnetic resonance imaging; MRS: magnetic resonance spectroscopy: VSGP: vertical supranuclear palsy.

## Competing interests

In the past 3 years, MTV has been an invited speaker in meetings organized and sponsored by Actelion, in postgraduate courses sponsored by Shire educational grants, and has served in an advisory board for Actelion. She has received occasional honoraria from Actelion and Shire.

## Appendix

Niemann-Pick diseases support groups and corresponding websites

1. Specific Niemann-Pick diseases support groups:

**UK**: Niemann-Pick disease Group (UK) http://www.niemannpick.org.uk

**USA and Canada**: National Niemann-Pick disease Foundation (USA): http://www.nnpdf.org; "Canadian chapter": http://www.nnpdf.ca Ara Parseghian Medical Research Foundation : http://www.parseghian.org

**Germany**: Niemann-Pick Selbshilfegruppe Deutschland : http://www.niemann-pick.de

**Spain **: Fundacion Niemann-Pick de España : http://www.fnp.es

**Italy**: Associazione Italiana Niemann-Pick: http://www.niemannpick.org

**Australia**: Australian NPC disease Foundation: http://www.npcd.org.au

**Argentina**: Asociacion Niemann-Pick Argentina: http://www.npc.org.ar

**Poland **: Stowarzysenie Chorych na NPC

2. Support groups for Lysosomal Diseases or Inborn Errors of Metabolism with a specific Niemann-Pick subgroup:

**France **(with antennas in French speaking areas of Belgium and Switzerland): Vaincre les Maladies Lysosomales http://www.vml-asso.org

**The Netherlands **:Volwassenen Kinderen en Stofwisselingsziekten : http://www.stofwisselingsziekten.nl
